# Hypertensive Crisis in Pediatric Patients: An Overview

**DOI:** 10.3389/fped.2020.588911

**Published:** 2020-10-20

**Authors:** Rupesh Raina, Zubin Mahajan, Aditya Sharma, Ronith Chakraborty, Sarisha Mahajan, Sidharth K. Sethi, Gaurav Kapur, David Kaelber

**Affiliations:** ^1^Department of Nephrology, Akron Children's Hospital, Akron, OH, United States; ^2^Akron Nephrology Associates/Cleveland Clinic Akron General Medical Center, Akron, OH, United States; ^3^Department of Internal Medicine, Northeast Ohio Medical University, Rootstown, OH, United States; ^4^Cleveland Clinic Akron General Medical Center, Akron, OH, United States; ^5^Pediatric Nephrology and Pediatric Kidney Transplantation, The Medicity Hospital, Kidney and Urology Institute, Medanta, Gurgaon, India; ^6^Division of Pediatric Nephrology and Hypertension, Children's Hospital of Michigan, Wayne State University School of Medicine, Detroit, MI, United States; ^7^Departments of Pediatrics, Internal Medicine, Population and Quantitative Health Sciences, Center for Clinical Informatics Research and Education, Case Western Reserve University and Metro Health System, Cleveland, OH, United States

**Keywords:** hypertensive emergency, hypertensive urgency, hypertensive crisis, management, acute severe hypertension

## Abstract

Hypertensive crisis can be a source of morbidity and mortality in the pediatric population. While the epidemiology has been difficult to pinpoint, it is well-known that secondary causes of pediatric hypertension contribute to a greater incidence of hypertensive crisis in pediatrics. Hypertensive crisis may manifest with non-specific symptoms as well as distinct and acute symptoms in the presence of end-organ damage. Hypertensive emergency, the form of hypertensive crisis with end-organ damage, may present with more severe symptoms and lead to permanent organ damage. Thus, it is crucial to evaluate any pediatric patient suspected of hypertensive emergency with a thorough workup while acutely treating the elevated blood pressure in a gradual manner. Management of hypertensive crisis is chosen based on the presence of end-organ damage and can range from fast-acting intravenous medication to oral medication for less severe cases. Treatment of such demands a careful balance between decreasing blood pressure in a gradual manner while preventing damage end-organ damage. In special situations, protocols have been established for treatment of hypertensive crisis, such as in the presence of endocrinologic neoplasms, monogenic causes of hypertension, renal diseases, and cardiac disease. With the advent of telehealth, clinicians are further able to extend their reach of care to emergency settings and aid emergency medical service (EMS) providers in real time. In addition, further updates on the evolving topic of hypertension in the pediatric population and novel drug development continues to improve outcomes and efficiency in diagnosis and management of hypertension and consequent hypertensive crisis.

## Introduction

Hypertensive crisis can be a source of great harm to the pediatric population. With its potential for rapidly progressing end-organ damage, hypertensive crisis should be promptly identified and concomitantly treated. Hypertensive crisis is defined as an acute episode of severely elevated blood pressure with potential for end-organ damage, often exceeding the limits known for stage II hypertension (1–7). While there are no specific cutoffs in terms of blood pressure for hypertensive crisis in pediatric patients (Table 1), as there are in the adult population, hypertensive crisis is primarily a clinical diagnosis which should be suspected in any pediatric patient with blood pressure at or exceeding the limits of stage II hypertension. Though the exact prevalence of hypertensive crisis is not yet known, the potential dangers of hypertensive crisis, in the form of organ damage, are well-known (8, 9). Hypertensive crisis can be subcategorized as hypertensive urgency, in which there are no signs of end-organ damage, and hypertensive emergency, in which signs of end-organ damage are present. While hypertensive crisis may be a result of primary hypertension, secondary causes are most often found in pediatric patients with hypertensive crisis. The management of patients suspected of being in hypertensive crisis revolves around decreasing blood pressure in the acute setting while identifying and treating the underlying cause in order to prevent organ damage from occurring (10–14). Here we review the current understanding of epidemiology, pathophysiology, diagnosis, and management of hypertensive crisis in a pediatric population as well as recent updates and developments that are on the horizon.

**Table 1:** Guidelines for diagnosis and staging of hypertension in children.

**Stages**	**Hypertension guidelines**										
	**AAP fourth report (2004) (mm Hg) (1)**	**AAP 2017 (mm Hg) (****2****)**		**ESH 2016 (mm Hg) (****3****)**		**ESC/ESH 2018 (mm Hg) (****4****)**		**SHMS 2018 (mm Hg) (5)**	**Malaysian society of HTN, malaysian academy of medicine (2018) (mm Hg) (****6****)**		**Canadian journal of cardiology 2018 (mm Hg) (7)**
Age		Age 1–12	Age 13–17	Age 0–15	Age ≥1–6	Age 0–15	Age ≥1–6		Age 1–13	Age> 13	
Normal BP	<90th percentile	<90th percentile	<120/80	<90th percentile	<130/8 5	<90th percentile	120–129/80–84	<90th percentile	<90th percentile	<120/80	<95th percentile
Elevate D BP	≥90th to <95th percentile	≥90th to <95th percentile or 120/80 to <95th percentile	120–129/ <80	>90th to <95th percentile	130–139/80–85	>90th to <95th percentile	130–139/85–89	90th to <95th percentile or if BP >120/80			
HTN	≥95^th^ percentile	≥95th percentile	>130/80	≥95th percentile	≥140/90	≥95th percentile	≥140/90	≥95th percentile		≥130/80	≥95th percentile
Stage I HTN	95th to 99th percentile and 5 mm Hg	≥95th to <95th percentile+1 2 mm Hg or 130–139/80–89	130–139/80–89	95th to 99th percentile and 5 mmHg	140–159/90–99	95th to 99th percentile and 5 mmHg	140–159/90–99	95th-99th percentile + 5 mm Hg	≥95^th^ to <95th percentile +12 mmHg or 130–139/80–89	130/80–139/89	Between 95th to 99th percentile plus 5 mm Hg
Stage II HTN	>99th percentile plus 5 mm Hg	≥95th percentile +12 mm Hg or >140/90	≥140/90	>99 percentile + 5 mm Hg	160–179/100–109	>99 percentile + 5 mm Hg	160–179/100–109	>99th percentile +5 mm Hg	≥95th percentile +12 or ≥140/90 mm Hg	≥140/90	>99th percentile + 5 mm Hg
Stage III HTN	N/A	N/A	N/A	N/A	N/A	N/A	≥180/≥110	N/A	N/A	N/A	N/A

## Epidemiology

The epidemiology of hypertensive crisis in children is difficult to pinpoint due to variations in diagnostic criteria and paucity of relevant literature. In a recent survey conducted by the National Health and Nutrition Examination Survey (NHANES) in preadolescent and adolescent patients, the morbidity of hypertensive crisis was found to be between 1 and 4% (15). Several retrospective studies conducted in the emergency room (ER) have shown the prevalence of hypertensive crisis among those presenting with HTN to range from 16 to 54% (16, 17). In contrast, a study conducted at a tertiary care referral center by Hari et al. showed that only 0.14% had hypertensive crisis with complications of end-organ damage during evaluation (17).

Similarly, a cross-sectional, single-center study performed in Houston, Texas, established the prevalence of hypertensive crisis among children to be as low as 0.6% (18). With the wide range of prevalence found for hypertensive crisis in the pediatric population, more multicentered studies are needed to accurately identify the true prevalence of hypertensive crisis in this population.

## Etiology

**Figure 1:**
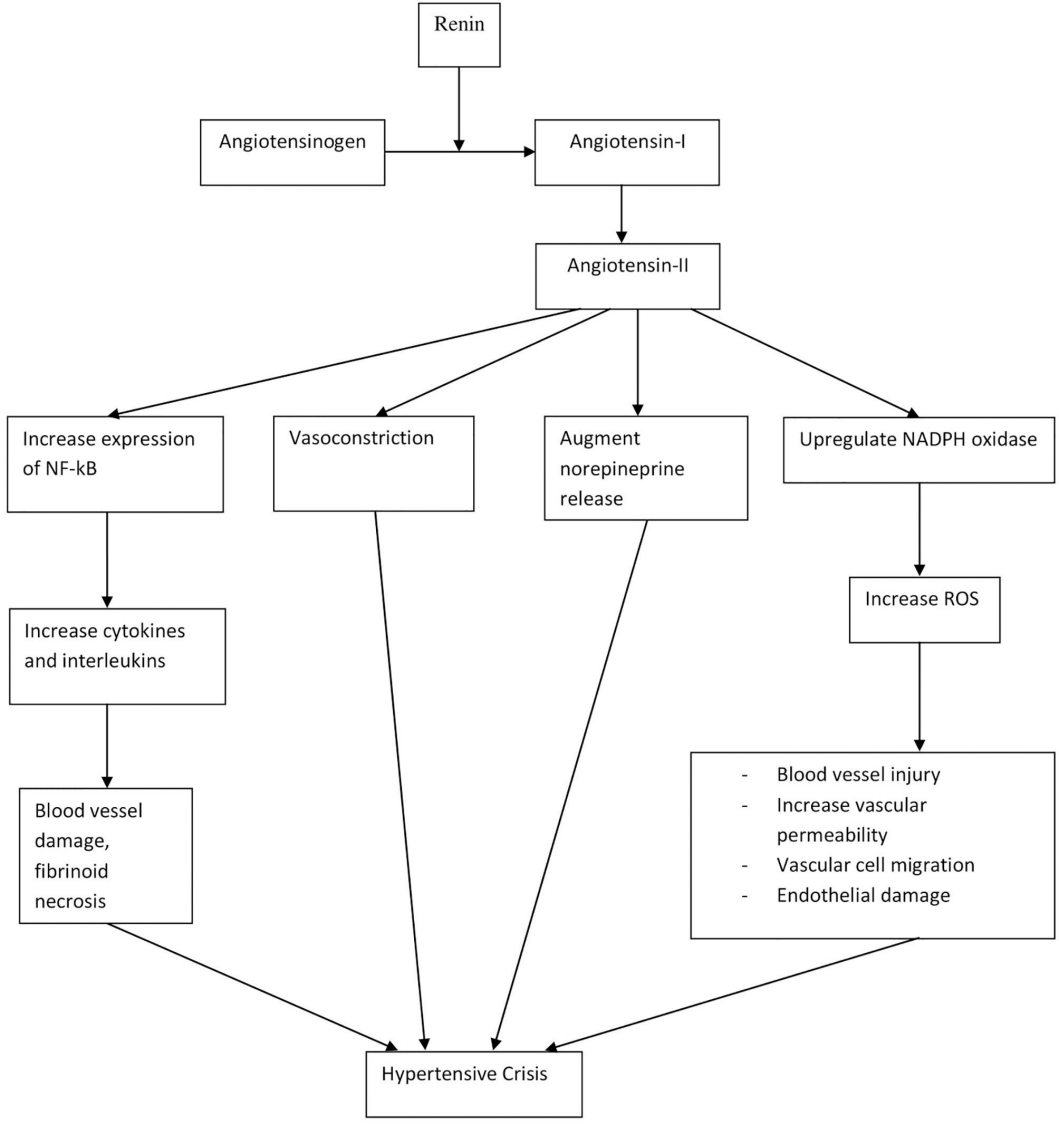
Pathophysiology of hypertensive crisis.

The causes of hypertensive crisis are similar to those of pediatric hypertension, both of which are multifactorial and influenced by the patient's age. In the pediatric population, especially at younger ages, 70–85% of hypertension is due to an underlying secondary cause. Of all the secondary causes contributing to hypertensive crisis, renal parenchymal diseases and coarctation of the aorta are the most frequent etiologies (19). Specifically, newborns are often afflicted with renal artery thrombosis/stenosis, congenital renal malformations, and coarctation of the aorta, whereas children older than 6 years of age are often found having renal parenchymal disease and renal artery stenosis as secondary causes of hypertension (20). In the absence of renal and congenital cardiac causes, one must be cognizant of formerly unidentified endocrinologic causes. Endocrine pathologies such as pheochromocytoma, paragangliomas, and monogenic causes of hypertension (Figure 1), which encompass disorders of regulation of kidneys and adrenal glands, can all lead to secondary hypertension and consequent hypertensive crisis in the pediatric population. The burden of cases of acute hypertension among adolescents are the end-result of non-adherence to prescribed medications, abrupt withdrawal of antihypertensive medications, and substance abuse with cocaine and amphetamines, as well as over-the-counter agents containing phenylpropanolamine, pseudoephedrine, and non-steroidal anti-inflammatory drugs (21–27). Although primary HTN accounts for 90% of hypertension among adolescents, it seldom intensifies to crisis and therefore hypertensive crisis in the adolescent population is most often a consequence of secondary HTN (28–30). It is interesting to note that recent AAP guidelines excluded children with obesity for establishing age-, sex-, and height-associated cutoffs for diagnosing hypertension in the pediatric population. As obesity is associated with higher BP readings, it is possible that obese children or adolescents (>13 years cutoffs similar to adult) may present with BP > stage 2 cutoffs. In the absence of hypertensive emergency or signs and symptoms of end-organ damage, these patients will present unique diagnostic challenges and thus highlight the importance of obtaining a full workup in those suspected of hypertensive crisis.

## Pathophysiology

**Figure 2:**
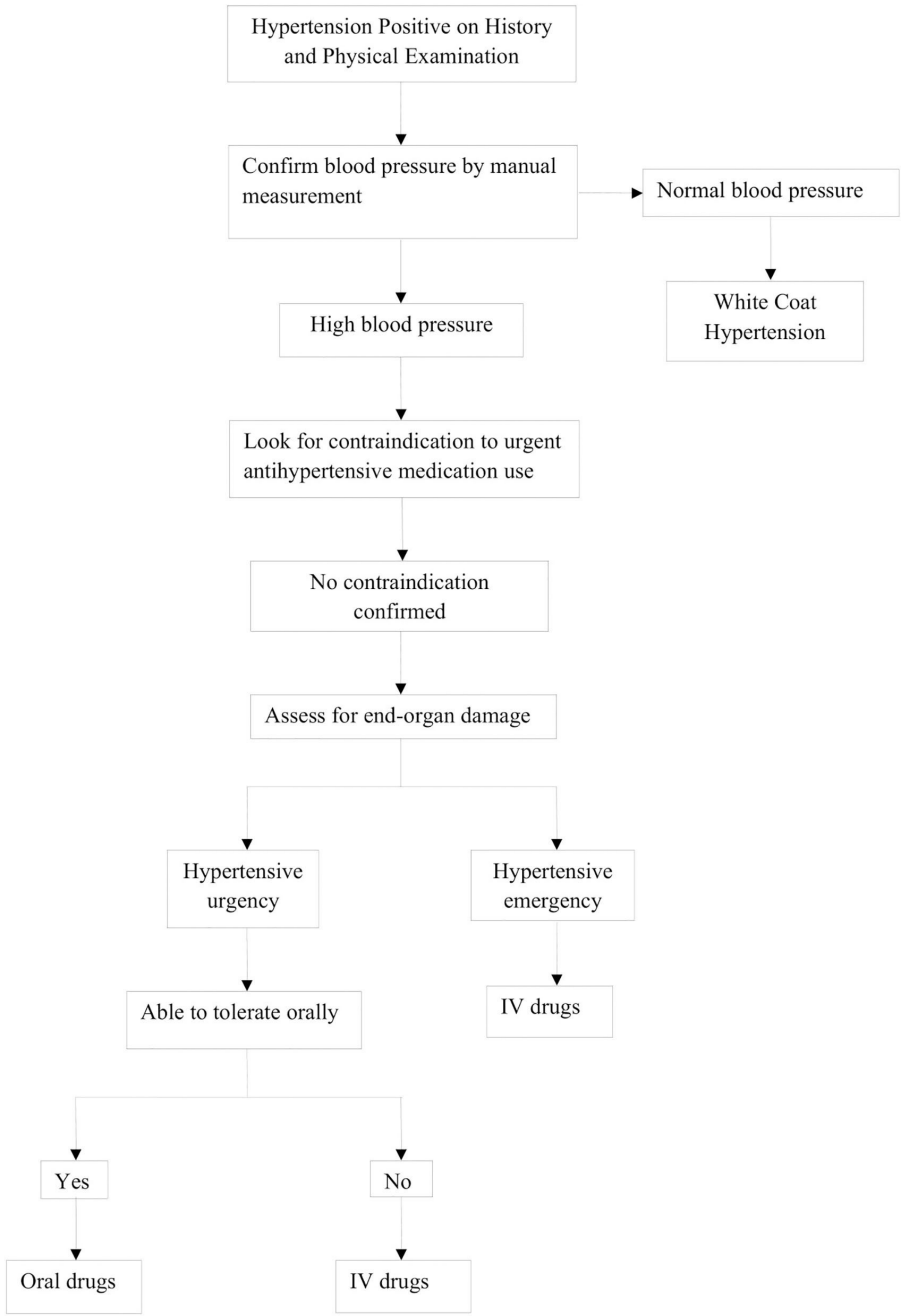
Guidelines for the evaluation of hypertensive crisis.

The pathophysiology and mechanisms contributing to elevated BP are similar in both hypertensive urgency and emergencies, although patients with hypertensive emergency have higher BP than urgency (9). Therefore, hypertensive crisis can be understood over a spectrum, with hypertensive urgency at the lower end, without any organ damage, and emergency at the higher end, with the presence of end-organ damage. Hypertensive crisis is the end result of a complex integration of many factors. While the pathophysiology of hypertensive crisis is dependent on each underlying cause, it often involves vasoconstriction and disruption of autoregulatory mechanisms in blood vessels (31). In addition, the renin–angiotensin–aldosterone system (RAAS), inflammatory mediators, and oxidative stress have all been implicated as shown in Figure 2 (32–37). Hypertensive crisis may also directly cause physical damage to blood vessels due to the stress exerted on wall of blood vessels, thus causing fibrinoid necrosis, endothelial damage, and activation of the coagulation cascade (37).

## Blood Pressure: Screening and Measurement

When considering the diagnosis of hypertensive crisis, it is of utmost importance to measure blood pressure in the acute setting. The American Academy of Pediatrics guidelines for hypertension from 2017 highlight important aspects of blood pressure measurement, including attention to cuff size as well as choosing the appropriate method of measurement, preferably auscultatory (manual) method over oscillometric (automatic) when possible (38, 39). As the optimal age for beginning blood pressure screening is currently not yet established, it is currently recommended that pediatric patients ages 3 and above have annual blood pressure measurements, as they can potentially detect asymptomatic hypertension and prevent progression to hypertensive crisis (38).

## Clinical Features

The clinical presentation of hypertensive crisis may be varied and often depends on the severity of end-organ damage. Identifying hypertensive crisis can thus be challenging as patients may present with non-specific symptoms that may be difficult to distinguish from other common illnesses. Hypertensive urgency, also known as acute severe hypertension, has a greater propensity to present with non-specific symptoms, such as irritability and poor feeding in infants and headache, nausea, fatigue, and dizziness in children and adolescents (40, 41). Hypertensive emergencies, on the other hand, may present with distinct signs of acute neurological, visual, cardiac, and renal damage. Of the known presentations of hypertensive emergencies, acute neurological signs are most common and are a result of disruption of the blood–brain barrier, insufficient oxygen delivery, and edema and microhemorrhages (42). These neurologic symptoms may be non-specific, with 55% of patients experiencing headaches, 46% with dizziness, and 36% with nausea/vomiting, as in hypertensive urgency, but can also present with further signs of neurologic damage, with 16% presenting with altered consciousness and 11–20% experiencing seizures (16, 43). A severe neurological complication of hypertensive crisis in children is posterior reversible encephalopathy syndrome (PRES), which principally involves damage to the occipito-parietal white matter and may spread to the basal ganglia, cerebellum, and brainstem. PRES can have a wide variety of presentations, ranging from minor symptoms such as headache, nausea, to major symptoms, such as altered mentation, seizures, focal neurologic deficit, or blindness (44, 45).

Hypertensive emergencies may also cause acute visual changes in the form of acute ischemic optic neuropathy, papilledema, hemorrhages, and cortical blindness (9). Williams et al. found that 18% of the children involved in the workup of severe renovascular HTN had retinal damage (46). Still et al. found that 36% of children with severe hypertension had papilledema in contrast to the study results by Wu et al., where 3.6% had visual disturbance (9, 47). Furthermore, hypertensive emergencies can cause cardiovascular remodeling in the form of left ventricular hypertrophy (LVH), which can consequently lead to congestive heart failure (CHF), manifesting with signs and symptoms such as dyspnea, chest discomfort, and gallop rhythm on auscultation. Several studies have shown that 13–26% of patients with hypertensive emergency have LVH (18, 43). Of note, several case reports have shown that hypertensive crisis in infants and neonates may paradoxically present with hypotension and cardiogenic shock (48, 49). In addition, it has been observed that hypertensive damage to the kidneys may manifest as hematuria, flank pain, and oliguria. Patients with unilateral renal artery stenosis may develop hyponatremic hypertensive syndrome (HHS), presenting with polyuria, polydipsia, and headaches as well as other neurological symptoms (50). By identifying the key clinical features of hypertensive emergency, one may adequately prepare for evaluation of such patients.

## Evaluation

**Figure 3:**
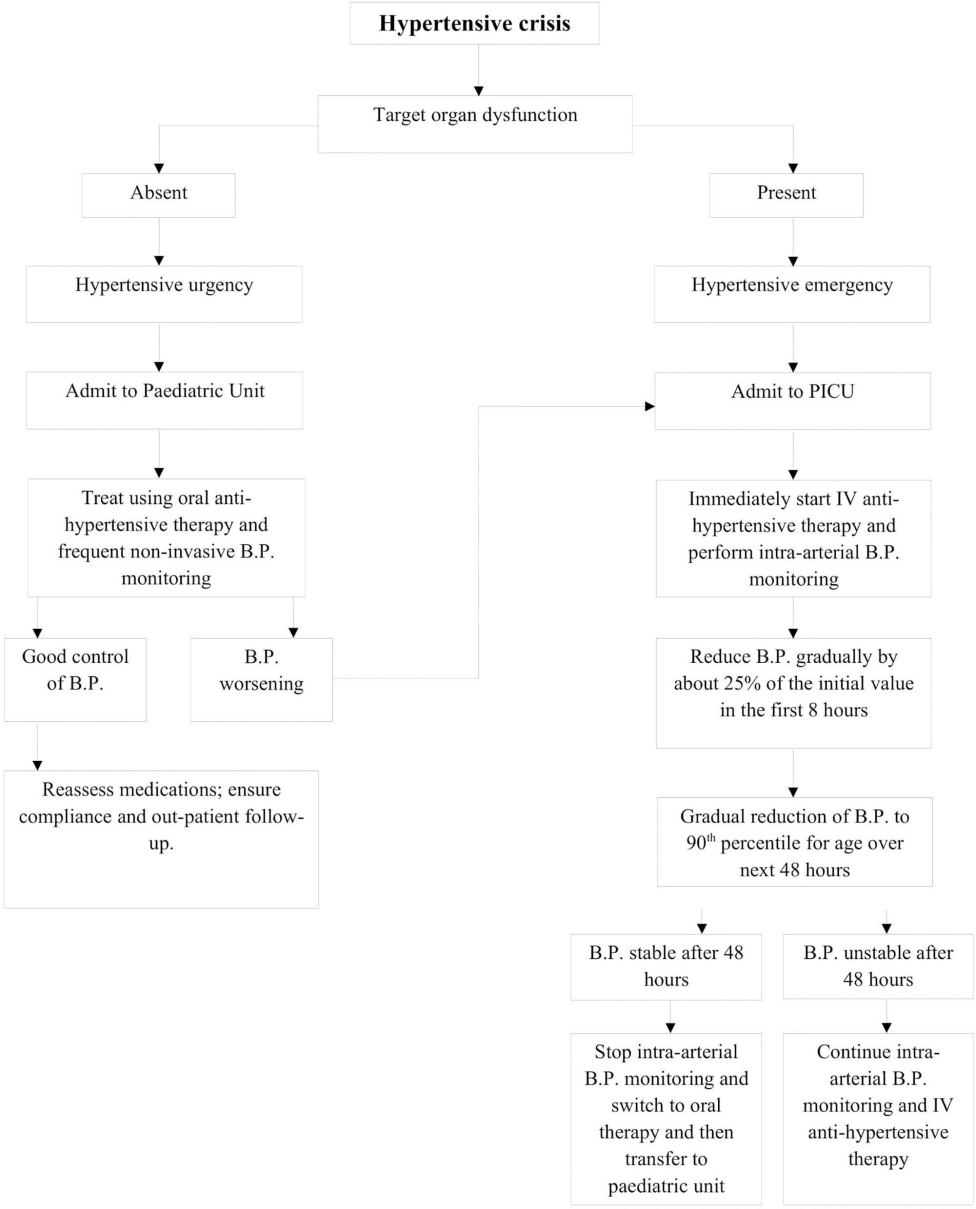
Management outline for hypertensive crisis.

Evaluation of hypertensive crisis begins with a stepwise, systematic protocol, as highlighted in Figure 3. This begins with measurement of vital signs, including BP, as well as the history and physical exam.

### History and Physical Exam

Obtaining a proper history and physical examination is very important in identifying the underlying causes of hypertensive crisis. As such, the history of a patient with hypertensive crisis should include a detailed account of all the symptoms, past medical history, and perinatal, nutritional, psychosocial, family, and medication history. The healthcare provider should also question the adolescents regarding the use of oral contraceptive pills, anabolic steroids, and recreational drugs (51). BP measurements are conducted manually or by automatic means; however, confirmation of abnormal values should be with auscultation. Initial evaluation of a patient should focus on assessment of end-organ damage during hypertensive emergencies. The systemic examination begins with assessment of the cardiovascular system for the presence of displaced apex beat, gallops, murmurs, and additional heart sounds, such as S3 and S4 or for lung crackles. The abdominal evaluation focuses on the presence of any bruit specifically indicative of renal vascular abnormalities, and signs of congestive hepatomegaly. The neurological evaluation should encompass the mental status exam, reflexes, vision, tone, and sensory or motor disturbances. Ophthalmoscopy is recommended to look for retinal vessel narrowing and papilledema (52). The summary of physical exam findings with their corresponding causes is shown in Table 2 (53–75).

**Table 2:** Physical examination findings in hypertensive crisis.

**Physical exam in hypertensive crisis**	**Etiology**	**Sensitivity**	**Specificity**
**Vitals**			
Tachycardia	Pheochromocytoma (53)	89%	67%
	Hyperthyroidism (54)	96%	72%
Upper to lower BP gradient	Coarctation of aorta (55)	41.30%	81.50%
Elevated BP	Hypertension (56)	34–69%	73–92%
**General**			
Short stature	Turners syndrome (57)	97%	96%
Truncal obesity	Cushing syndrome (58)	27%	95.20%
Weight loss	Hyperthyroidism (54)	50%	90%
**Head and Neck**			
Retinal arteriolar narrowing	Hypertensive retinopathy (59)	19%	89%
Retinal AV nicking	Hypertensive retinopathy (59)	3%	98%
Exophthalmos	Hyperthyroidism (54)	55%	86%
Thyroid enlargement	Hyperthyroidism (54)	56%	72%
**CVS**			
Pericarditis	SLE (60)	56%	86%
Murmurs	Coarctation of aorta (55)	92%	88%
Apical heave	LVF (61)	56%	91%
**RS**			
Adventitial lung sounds/SOB	LVF (62)	60%	78%
**Abdominal**			
Bruits	Renal artery stenosis (63)	63%	90%
**CNS**			
AMS	HTN encephalopathy (64)	12.5–40%	
Seizure	HTN encephalopathy (64)	8.3–14.3%	
**Skin**			
Striae	Cushing syndrome (58)	95%	100%
Flushing/diaphoresis/pallor	Hyperthyroidism (54)	89%	67%
	Pheochromocytoma (53)	57%	96%
Malar rash	SLE (60)	17%	100%
Heat intolerance	Hyperthyroidism (54)	92%	100%
**Extremities**			
Odema	LVF (61, 62)	51%	76%
Joint pain	SLE (60)	86%	37%
**Investigations**			
Serum creatinine	Renal failure (65)	12%	99.90%
Urine dipstick	Hematuria, proteinuria (65)	100%	29.70%
Urine toxicology (cocaine/amphetamine)	Detect substance abuse (66)	48%/18.2%	94.2%/98.8%
ECG	LVH (67)	67%	93%
CXR	CHF (68)	71%	92%
ECHO	LVH (69)	68–76%	48–77%
Serum cortisol	Cushing's syndrome (70)	98.6%	90.6%
Plasma metanephrine and normetanephrine	Pheochromocytoma (71)	100%	94%
Plasma norepinephrine and epinephrine	Pheochromocytoma (71)	92%	91%
Urine metanephrine and nor metanephrine	Pheochromocytoma (71)	100%	95%
Urine norepinephrine and epinephrine	Pheochromocytoma (71)	100%	83%
Urine vanillylmandelic acid	Pheochromocytoma (71)	63–75%	94%
Renal angiography (MRA)	Renal artery stenosis (72–74)	64–93%	72–97%
Renal angiography (CT)	Renal artery stenosis (72–74)	64–93%	62–97%
Captopril renal scan	Renal artery stenosis (72–74)	52–93%	63–92%
Plasma aldosterone/renin ratio	Hyperaldosteronism (75)	98.9%	78.9%

### Imaging and Laboratory Workup

After a thorough clinical assessment, the initial workup of a patient with hypertensive crisis should include a complete blood count (CBC), serum chemistry, and urinalysis, while further workup can be classified by organ system being investigated: cardiac, renal, adrenal, and neurological. For cardiac examination, the electrocardiogram (ECG), echocardiography (ECHO), and chest X-ray (CXR) have not been useful due to low sensitivity of these tests to detect cardiopulmonary damage (67–69). Investigating renal and adrenal causes includes obtaining serum cortisol, renin, and aldosterone levels, as well as urine catecholamine levels (75). Renal imaging using computed tomography angiography (CTA) or magnetic resonance angiography (MRA) may be used as an investigative tool for evaluation of renal artery stenosis, but lacks sensitivity for detection of intraparenchymal stenotic changes (74, 76). Specialized tests such as computerized tomography (CT)/magnetic resonance imaging (MRI) scan of the brain may be required to evaluate the extent of neurologic involvement, including evaluation for intracranial bleeding and PRES (77). Finally, in cases with a high index of suspicion for drug abuse, urine toxicology should be conducted. By performing a full and thorough workup, the clinician can pinpoint the cause and move forward to management of hypertensive crisis.

## Management

### General Guidelines

After evaluation, management of a pediatric hypertensive crisis, as outlined in Figure 3, begins with simultaneous rapid acting antihypertensives, barring any contraindications to their use. The principal goal of management is to gradually reduce BP and prevent end-organ dysfunction. In children and adolescents diagnosed with hypertensive crisis, the treatment goal with non-pharmacologic and pharmacologic therapy should be a reduction in SBP and DBP to <90th percentile and <130/80 mm Hg in adolescents ≥13 years of age (12, 13). The rate of BP reduction should be 25% over a period of 6–8 h, which is gradually reduced to normal over 24–72 h since sudden, drastic reductions in blood pressure can itself contribute to organ damage secondary to ischemia (11, 12, 20). The various therapeutic drugs have been outlined in Table 3 and are described in greater detail in further sections. It is important to note the importance of blood pressure monitoring throughout the treatment course due to the potential for precipitous drops in blood pressure, which are best measured with intra-arterial blood pressure monitoring. This method is preferred for critically ill children as well as when using drugs with potential to cause severe hypotension.

**Table 3:** Characteristics of medications for management of hypertensive crisis.

**Drug**	**Class**	**FDA approval**	**Onset (min)**	**Duration (hour)**	**Route**	**Dose**	**ADR**	**C/I**	**Characteristics**
**Parenteral therapy**									
Labetalol	α + β blocker	No	2–5	2–12	IV bolus or Infusion	0.2–1 mg/kg/dose, up to 40 mg/dose; infusion 0.25–3 mg/kg/hr	Bradycardia, heart block, aortic dissection, hyperkalemia, hypoglycemia	Asthma, insulin dependent Diabetics, heart failure	Do not withdraw abruptly, metabolized by hepatic glucuronidation, safe in renal dysfunction
Nicardipine	Calcium channel blocker	No	10	<8	IV bolus or infusion	Bolus: 30 μg/kg up to 2 mg/dose; infusion: 0.5–4 μg/kg/min	Hypotension, tachycardia, headache, thrombophlebitis	Hypersensitivity	Use with caution in hepatic dysfunction
Hydralazine	Arterial vasodilator	No	5–20	1–4	IV/IM bolus	IV: 0.2–0.6 mg/kg, maximum single dose 20 mg. Repeat bolus q 4 h	May cause reflex tachycardia, fluid retention, or headaches	Lupus, Hypersensitivity	Can cause drug-induced lupus. Duration depends on rate of acetylation. Metabolized mainly in liver and kidneys
Esmolol	Beta- 1 blocker	No	2–10	10–30 (min)	IV infusion	100–500 μg/kg/min, up to 1,000 μg/kg/min	Bradycardia	Asthma, heart failure	Very short-acting. Counteracts reflex tachycardia
Sodium Nitroprusside	vasodilator	Yes 11/22/2013	2–10	1–10 (min)	IV infusion	0.5–10 μg/kg/min	Cyanide toxicity and thiocyanate toxicity, headache palpitations	Head injury as it can increase cerebral blood flow and ICP	Monitor cyanide levels with prolonged use (>48 h) or in hepatic or renal failure, or co-administer with sodium thiosulfate. Needs constant BP monitoring with arterial line. Can be used in HTN crisis with CHF
Fenoldopam	Dopamine receptor agonist	Yes 04/01/2004	10	1	IV infusion	0.2–0.8 μg/kg/min	Reflex tachycardia	Xerostomia, Hypersensitivity	It develops tolerance after 48 h. It increases renal perfusion, promotes natriuresis, improves urine output.
Clevidipine	Calcium channel blocker	No	2–4	5–15 (min)	IV infusion	0.5–3.5 μg/kg/min	Reflex tachycardia, headache, hypotension	Egg and soya allergy, Lipid disorders	Limited pediatric data on dosing, ultra-short acting
Phentolamine	α-blocker	No	Immediate	15–30 (mi n)	IV bolus	0.05–0.1 mg/kg/dose, up to 5 mg	Tachycardia	Hypersensitivity	Can be used in Pheochromocytoma, paraganglioma, cocaine/amphetamine abuse
Furosemide	Loop diuretic	No	Within minutes		IV bolus	0.5–5 mg/kg per dose	Hypokalemia, hyperuricemia Pancreatitis	Gout	Used in patients with HTN crisis due to glomerulonephritis, CHF
Enalapril	ACE inhibitor	Yes 09/08/2014	15–30	6–12	IV bolus	5–10 μg/kg/dose up to 1.2 mg/dose	Hyperkalemia, dry cough, hypotension,	Acute renal failure, angioedema	Renoprotective
**Oral therapy**									
Isradipine	Calcium channel blocker	No	60	3–8	PO	0.05–0.1 mg/kg/dose up to 5 mg/dose	Hypotension, headache, flushing	With azoles, Hypersensitivity	Concurrent use of azole antifungals leads to hypotension
Clonidine	Central alpha agonist	No	15–30	6–8	PO	0.05–0.1 mg/dose, may be repeated up to 0.8 mg total	Dry mouth, sedation, Rebound hypertension	Hypersensitivity	Safe in renal failure
Minoxidil	Arterial vasodilator	No	30–60	8–12	PO	0.1–0.2 mg/kg/dose up to 10 mg/dose pericardial effusion	Hypotension, fluid retention, hirsutism, hypertrichosis, pericardial effusion	Pericardial effusion Hypersensitivity	Most potent oral vasodilator, long acting, titrated slowly, No Compoundable solution available
Nifedipine	Calcium channel blocker	No	20–30	3–8	PO/SL	0.1–0.25 mg/kg/dose	Tachycardia, headache, MI	MI Hypersensitivity	Can cause precipitous drop in blood pressure

Intra-arterial BP monitoring is done through catheterization of the radial artery, which allows for monitoring of subtle changes in the blood pressure (2). Although it allows for highly accurate BP monitoring, it has been associated with increased risk of distal ischemia, vasospasm, and systemic infection, all of which can be limited with appropriate equipment use (78).

### Parenteral Therapy

Parenteral therapy is crucial to the management of hypertensive emergency, while cases of hypertensive urgency can be managed with either IV or rapid-acting oral antihypertensives. Among the most commonly used first line IV agents is labetalol, a combined alpha and beta blocker which acts both by reducing peripheral vascular resistance and through its negative chronotropic effect. Although it should be avoided in those with asthma and heart failure due to the beta-blocking effect of bronchoconstriction, it can be used in those with renal dysfunction, as it is hepatically metabolized. However, Thomas et al. showed that a concomitant traumatic brain injury can be a contraindication for labetalol, as it has been associated with increased rates of hypotension in this patient population (79). Similarly, esmolol is a rapid-acting beta-1 blocker that is ideal for critically ill patients with multiorgan failure. Esmolol is preferentially used in cases of hypertensive crisis accompanying repair of congenital heart disease (12). Nicardipine, another first-line IV agent, is a potent and rapid-acting dihydropyridine calcium channel blocker (CCB) that decreases blood pressure via decreasing peripheral vascular resistance. Nicardipine can be administered as continuous infusion or bolus therapy and is preferentially given via central access due to risk for thrombophlebitis with peripheral use (80). Finally, clevidipine is an ultrashort-acting IV CCB with a rapid onset, which causes arteriolar vasodilation and has an added advantage of a simplified dose titration due to rapid inactivation by tissue and blood esterase but is absolutely contraindicated in patients with egg and soy allergies as well as in those with lipid disorders (81). It has negligible negative inotropic or chronotropic effects on the heart.

The vasodilators are also effective and rapid-acting agents that are used in management of hypertensive crisis. Sodium nitroprusside is a first-line agent from this group with direct arterial and venous smooth muscle relaxant actions and is frequently used due to its ease of titration to prevent fluctuations in BP, its short half-life, and therefore, the rapid onset and termination of effects. However, it has potential to induce methemoglobinemia as well as cyanide and thiocyanate toxicities (82). Another commonly used vasodilator is hydralazine, an arterial dilator that is used due to its rapid onset while continuous infusion with IV agents is prepared. In hypertensive emergencies, hydralazine decreases systemic venous resistance via inhibition of calcium-dependent adenosine triphosphatase, and phosphorylation in arteriolar smooth muscle. Hydralazine lacks the negative inotropic effect and causes reflex tachycardia by activation of the RAAS pathway, which can negate its antihypertensive effects (83). Although hydralazine and nitroprusside are the most used vasodilators, fenoldopam is another agent that can be used. With activity at the dopamine 1 receptor and α-adrenoreceptors, fenoldopam causes increases in renal blood flow and urinary flow, in addition to natriuresis. Fenoldopam can be used safely for hypertensive crisis in patients with concurrent renal dysfunction (84).

Some drugs, such as enalaprilat, phenoxybenzamine, doxazosin, and furosemide, are used only in specific situations. Enalaprilat is used for high-renin hypertension and is the only angiotensin-converting enzyme inhibitor available as an IV formulation. The adverse effects are due to the anti-renin properties and range from hyperkalemia to functional acute kidney injury (AKI), especially in patients with underlying chronic kidney disease, bilateral renal artery stenosis, or solitary kidney (85). Similarly, α adrenergic blockers such as phenoxybenzamine and doxazosin are specifically used in catecholamine-induced hypertension such as paragangliomas and pheochromocytoma (86). Comparatively, furosemide is a loop diuretic that causes natriuresis and diuresis and is effective in children with volume-dependent hypertension, such as with oliguric AKI, glomerulonephritis, or CHF. However, its repetitive use can develop hypokalemia or volume depletion; therefore, serum potassium levels and hydration status should be regularly monitored (87).

### Oral Therapy

The utility of oral agents in management of hypertensive crisis is limited to hypertensive urgency, where sufficient time is available for onset of oral therapy. Isradipine, the most used oral therapy, is a second-generation dihydropyridine CCB that antagonizes L-type calcium channels causing vasodilatation (88). A study conducted by Flynn and Warnick showed that 51.4% of patients achieved adequate BP reduction with the use of isradipine (89). Of note, Miyashita et al. demonstrated azole antifungals as a contraindication of isradipine use, with all patients studied having experienced severe hypotension due to inhibition of metabolism of isradipine via CYP3A/4 (90). In addition, clonidine is another orally used antihypertensive drug that activates alpha 2-adrenergic receptors and decreases central sympathetic tone, thereby causing vasodilation. The biggest drawback of clonidine use is its tendency to cause rebound hypertension (91). In contrast, nifedipine and minoxidil are two orally acting agents associated with propensity to cause severe hypotension. Nifedipine is historically the shortest-acting CCB and often causes unpredictable reduction of BP leading to complications, such as cerebral ischemia or ventricular arrhythmia (92). Similarly, minoxidil causes predominant arteriolar dilatation by acting as a potassium channel opener without affecting the venous circulation. It has a very potent BP-lowering effect in all forms of hypertension, even in those that are refractory to other antihypertensives including volume hypertension in hyperhydrated dialyzed children (93). Protracted minoxidil use has been associated with hirsutism, severe hypotension, and possible pericardial effusion due to salt and water retention effects, which often necessitates use of furosemide diuretic (94).

### Management of Common Underlying Conditions

After discussion of the general guidelines of management, it is important to focus attention on management in those with underlying conditions, which often exacerbate to hypertensive crisis in pediatric patients and require specific protocols for management, as highlighted below.

#### Aortic Coarctation

Aortic coarctation is among the most common secondary cause of hypertensive crisis in the pediatric population. As such, it is crucial to understand its management, which includes the use of the beta-blocker esmolol as the drug of choice for infants and children with aortic coarctation. In a study performed by Weist et al., esmolol lowered BP effectively in 95% of hypertensive children aged 1 month to 12 years (95). In addition, it has been useful to counteract paradoxical hypertension emerging after repair of aortic coarctation. In a study conducted by Dittrich et al., esmolol was more efficacious than sodium nitroprusside for control of paradoxical HTN whereas another study by Tabbutt et al. showed safety of esmolol for paradoxical HTN with no significant difference with the use of variables doses (125, 250, 500 μg/kg/min) (96, 97). Although such measures aid in controlling hypertension due to aortic coarctation, the definitive treatment of coarctation of aorta often involves surgical intervention (98, 99).

#### Renovascular Hypertension, Renal Parenchymal Diseases, and AKI

Renal diseases, namely, renovascular and renal parenchymal diseases, are among the leading secondary causes of hypertensive crisis in the pediatric population (19). In patients with renal disease leading to hypertensive crisis, the treatment often includes angiotensin-converting enzyme (ACE) inhibitors, beta blockers, and diuretics. In cases of unilateral renal artery stenosis, ACE inhibitors can safely be used, although they are not recommended in cases of bilateral renal artery stenosis or solitary functioning kidney (100). Beta blockers may also safely be used in patients with both unilateral and bilateral renal artery stenosis due to their ability to reduce renin release from the kidneys (40). Additionally, fluid overload from renal parenchymal diseases and AKI may contribute to secondary hypertensive crisis. Therefore, loop diuretics such as furosemide are the mainstay of management for both glomerulonephritis and AKI since they counteract the sodium and water retention associated with progressive glomerular damage (51). A prospective study conducted by Pruitt et al. on 25 patients with AKI demonstrated complete safety and efficacy with both oral and IV forms of furosemide with dosage ranging from 1 to 5 mg/kg (87). In furosemide refractory cases, dialysis may help with management of fluid overload. IV calcium channel blockers such as nicardipine and clevidipine are potential alternatives for hypertension management in patients with AKI (51). Surgical measures such as revascularization, auto-transplantation, or nephrectomy (especially in small, poorly functioning kidneys causing hypertension) may be required for refractory cases (101).

#### Endocrinologic Neoplasms: Pheochromocytoma and Paraganglioma

Pheochromocytoma and paraganglioma are neuronal-based tumors contributing to hypertensive crisis via excessive secretion of catecholamines. The diagnosis of pheochromocytoma involves use of plasma or urine metanephrine assays, as shown in Table 2, followed by CT/MRI to identify tumor location. Additionally, it is important to evaluate for the presence of *Von–Hippel Lindau disease* (*VHL), neurofibromatosis-1(NF-1)*, and *rearranged during transfection (RET)* in cases of suspected pheochromocytomas and succinate dehydrogenase complex subunits (*SDHD, SDHB, SDHC*) gene mutations in paragangliomas. The Endocrine Society Clinical Practice Guideline recommends a three-stage process for management of pheochromocytoma and paragangliomas, with the goal of BP reduction to <50th percentile for age and weight (102). The preoperative stage begins 14 days prior to surgical intervention with the initiation of α-blockers, such as phenoxybenzamine or doxazosin at a dose of 0.2 mg/kg/day with addition of 6–10 g of salt and maintenance fluid to prevent hypotension (102–104). Beta blockers are used 3 days prior to counteracting the tachycardia originating from the catecholamine surge during intraoperative handling of tumor (102, 103). Use of β-blockers without α-blockers can prime to unopposed α-adrenergic stimulation and exacerbate rise in BP, and thus it is very important to have alpha-adrenergic blockade prior to initiation of beta blockers (104). A study conducted by Ludwig et al. recommends use of metyrosine, a tyrosine hydroxylase inhibitor, 1 day prior to prevent intraoperative fluctuations in BP. The surgical intervention involves resection of the tumor for which laparoscopic or adrenal sparing surgery is preferred with concurrent use of sodium nitroprusside and esmolol for intraoperative BP control. The postoperative stage encompasses cautious BP monitoring and use of IV fluids to prevent sudden hypotension (105).

#### Monogenic Hypertension

Monogenic hypertension encompasses a variety of etiologies that are associated with disruption of regulation of the kidneys and adrenal glands. As such, the focus of management is to counteract the effects of this disruption via various medical management, dependent upon each etiology as below (Table 4).

**Table 4:** Endocrine parameters and treatment of diseases with monogenic hypertension.

**Condition**	**Phenotype MIM**	**Gene/Locus MIM**	**Pattern of inheritance**	**Age**	**Potassium**	**Renin (PRA)**	**Aldosterone**	**Aldo: PRA ratio**	**Glucocorticoid Resp**.	**Mineralo corticoid receptor blocker Resp**.	**Treatment**
Liddle's	177200	600760	AD	Child Adult	N or ↓	↓	↓		-	-	Amiloride, Triamterene
Gordon's	145260	–	AD	Child Adult	N or ↑	↓	N or ↑		-	-	Triamterene
FMI	218030	614232	AR	Infant Child Adult	↓ (N)	↓	↓		-		Mineralocortico id rec, antagonist
H-P	605115	600983	AD	Child Adult	N or ↓	↓	↓			reversed	Amiloride, Triamterene Thiazide
GRA	10390	610613	AD	Infant Child	N or ↓	↓	↑	↑			Amiloride, Triamterene
FHD	605635	600570	AD	Adult	N or ↓	↓	↑	↑	-		Mineralocortico id rec, antagonist

*AME, apparent mineralocorticoid excess; H-P, hypertension exacerbated by pregnancy; GRA, glucocorticoid-remediable aldosteronism; FH II, familial hyperaldosteronism type II; CAH, congenital adrenal hyperplasia with 11- or 17-hydroxylase deficiency; FGR, familial glucocorticoid resistance; AD, autosomal dominant; AR, autosomal recessive; N, normal; ↓, decrease; ↑, increase; PRA, plasma rennin activity; Aldo, aldosterone; Aldo: PRA, ratio of aldosterone to PRA (<30 diagnostic if Aldo, in ng/dL, PRA in ng/mL/h); –r negative; +, positive*.

##### Primary hyperaldosteronism

Primary hyperaldosteronism is characterized by overproduction of aldosterone via the adrenal gland, leading to secondary HTN, hypokalemia, and sodium retention. The management of familial hyperaldosteronism type-I (FHT-I) involves the suppression of adrenocorticotropic hormone (ACTH) by use of glucocorticoids, ultimately preventing the secretion of aldosterone (106). If glucocorticoids fail, then mineralocorticoid receptor antagonists or sodium channel blockers can be used as alternatives (107). The mainstay of treatment for FHT-II, III, and IV are mineralocorticoid receptor antagonists or unilateral adrenalectomy depending on the severity of the HTN (108–110). Additionally, adrenal adenomas secreting aldosterone often require laparoscopic surgical excision for treating primary hyperaldosteronism (111).

##### Liddle syndrome

Liddle syndrome is a consequence of gain-of-function mutation of the gene encoding the three subunit channel proteins (alpha, beta, and gamma) of aldosterone-dependent epithelial sodium channel (ENaC) in the collecting ducts. These mutations inhibit degradation of ENaC and excessive sodium absorption culminating to hypertension, hypokalemia, metabolic alkalosis, and a low plasma renin and aldosterone. The diagnosis is verified by screening for mutations in the genes encoding the β and γ subunits ENaC. The treatment revolves around direct ENaC inhibition with sodium channel blockers such as amiloride or triamterene and a low sodium diet to counteract altered ENaC physiology (112).

##### Syndrome of Apparent Mineralocorticoid Excess (SAME)

SAME is an autosomal recessive disorder of 11-beta HSD2 deficiency characterized by severe HTN with target organ damage, hypercalciuria, nephrocalcinosis, hypokalemia, and metabolic alkalosis with low levels of plasma renin and aldosterone levels (110, 113). The biochemical diagnosis relies on high urine cortisol to corticosterone ratio (114). Spironolactone or eplerenone in addition to potassium supplements and sodium restriction is the mainstay of treatment for SAME (114–116).

Glucocorticoid therapy is acceptable to downregulate ACTH-mediated cortisol overproduction and its subsequent effects on mineralocorticoid receptors (116). Similar to SAME, licorice (glycyrrhetinic acid) consumption also causes HTN, sodium retention, and potassium loss due to inhibitory effects on 11-beta HSD2, and therefore, management of this condition is exactly parallel to SAME (117).

##### Congenital Adrenal Hyperplasia (CAH)

CAH, one of the frequent inborn functional defects of adrenal glands, has two types of enzyme defects which culminate to mineralocorticoid surplus mediated HTN: 11-β-hydroxylase deficiency and 17-α-hydroxylase deficiency (118). The 11-hydroxylase deficiency prevents hydroxylation of 11-deoxycortisol resulting in cortisol deficiency and prevents conversion of deoxycorticosterone to aldosterone and corticosterone. This leads to a surplus of 11-deoxycorticosterone, which causes hypertension and hypokalemia due to mineralocorticoid effects, in addition to virilization in these infants (119). Similarly, 17-α hydroxylase deficiency creates a diversion for flow of pregnenolone and progesterone toward mineralocorticoid production, excess of which leads to hypertension and hypokalemia (120). The central outline of management of congenital adrenal hyperplasia is to supplement glucocorticoids which downregulate ACTH secretion and suppress steroid synthesis. Spironolactone, amiloride, and calcium channel blockers may be added to counteract HTN and genital abnormalities in females may necessitate surgery (115, 121).

#### Cushing's Syndrome

Cushing's syndrome is characterized by excessive production of glucocorticoids, which potentiate the effect of catecholamines on blood vessels, thus causing hypertensive crisis. When suspecting Cushing's syndrome-induced hypertensive crisis, which may also present with signs of excess cortisol such as central obesity, moon facies, and hirsutism, it is important to identify the source of hypercortisolism through diagnostic testing with a 24-h urine cortisol level or low-dose dexamethasone suppression testing. The treatment often involves the use of metyrapone, an 11-beta hydroxylase inhibitor which suppresses cortisol production. A study conducted by Nieman et al. demonstrated improved efficacy with the combination of metyrapone and ketoconazole (122). For central hypercortisolism, also known as Cushing disease, hypophysectomy with or without radiation therapy is preferred. For primary hypercortisolism, excision of the mass and in some cases adrenalectomy may be required (122, 123).

#### Adrenal Incidentaloma

Adrenal incidentaloma is a tumor of adrenal glands detected incidentally during abdominal imaging for other reasons. The incidentaloma may have a varied presentation from completely asymptomatic, virilization to hypertensive crisis secondary to pheochromocytoma, hypercortisolism, or primary hyperaldosteronism due to excess production of catecholamines, cortisol, and aldosterone, respectively. The workup of incidentalomas includes a detailed history and physical, hormonal screening tests, overnight dexamethasone suppression tests, plasma/urine metanephrine, plasma aldosterone, and renin levels. Imaging tests utilized may be CT/MRI abdomen, and fine needle aspiration biopsy may be required to rule out malignancy. The management of incidentaloma is primarily surgical excision especially if >4 cm with appropriate BP control using antihypertensive medications (123, 124).

#### Hypertension Management in Chronic Kidney Disease (CKD)

Patients with CKD can develop hypertensive crisis due to alteration of the RAAS pathway. These patients have inappropriately normal or high renin levels due to abnormal renin secretion from poorly perfused areas. This ultimately contributes to angiotensin II-mediated vasoconstriction and aldosterone-mediated volume retention leading to hypertensive crisis (125, 126). In ~75% of children with CKD stages 2–4, BP can be alleviated below the 95th percentile with monotherapy, while 50–60% of children require gradually titrated multidrug therapy. ACE inhibitors or angiotensin receptor blockers are first-line agents used due to their efficacy for BP reduction and renoprotective effects and are especially preferred in proteinuria patients (125, 126). Although ACE inhibitors are preferred for hypertension due to CKD, crisis episodes may require IV nicardipine therapy for rapid BP control since it has been used safely in children (127).

## Future Directions: Diagnosis, Technological Advances, and Medications

Diagnosis of hypertension in the pediatric population can at times be a complicated process and is often dependent upon which guidelines are being used. The Canadian Journal of Cardiology recently published the 2020 revised guidelines for diagnosing pediatric hypertension, with the goal of simplicity in mind. Based on the results of the Bogalusa Heart Study, the Canadian Journal of Cardiology recommends diagnosis of hypertension for pediatric patients aged 6–11 to be >120/80, whereas patients aged 12–17 have the new cutoff of >135/85, as the above cutoffs where shown to equally predict long-term outcomes as opposed to previous diagnostic criteria (128). As the above study shows equal predictive value with the simplified cutoffs, revisions in diagnostic criteria among other diagnostic guidelines may also be of benefit in the diagnosis of both hypertension and hypertensive crisis.

With the advent of telehealth, capabilities across the healthcare field have exponentially increased. As such, utilization of telehealth and other new technological advances can broaden and potentially increase clinicians' reach outside of hospitals and care centers. This newfound reach may be especially beneficial in cases of emergencies, including hypertensive crises. Brokmann et al. studied the effect of real-time vital data transmission as well as video capabilities to hospital physicians in cases of pre-hospital hypertensive crisis. These telemedicine capabilities were found to be beneficial in cases of pre-hospital hypertensive crisis, with less pronounced blood pressure drops and increased adherence to clinical guidelines in telemedically guided treatment by EMS personnel (129). These capabilities could be expanded to treatment of pediatric patients suspected of hypertensive crisis prior to arrival to the hospital, potentially preventing end-organ damage, and life-long complications of hypertensive emergency.

In addition to increases in telehealth capabilities, there have also been rising levels of primary hypertension in the pediatric population on a global scale (130). Consequently, primary hypertensive-induced hypertensive crisis may potentially become a greater proportion of hypertensive crisis, and thus adequate preventive measures, including lifestyle changes, as well as screening and management may be more important than ever before. In addition, this increase in primary hypertension around the world may warrant further epidemiological investigation to study if the proportion of primary hypertensive-induced hypertensive crisis is increasing.

In conjunction with the rise in primary hypertension in pediatric patients, there has also been a global increase in the prevalence of treatment-resistant hypertension (131). As such, novel treatment methods are needed to manage such conditions and prevent further development of hypertensive crisis. Among the novel medications on the horizon is firibastat, a first-in-class brain aminopeptidase A inhibitor, which leads to decreased levels of angiotensin III. Firibastat not only has shown promise in management of treatment-resistant hypertension but also has been shown to be safe as well (132). While the prevalence of treatment-resistant hypertension is not yet established in the pediatric population, much like hypertensive crisis, firibastat may play a role in the management of treatment-resistant hypertension and hypertensive crisis in pediatric patients in the near future.

## Conclusion

Hypertensive crisis can result in significant harm to the pediatric population. Not only is it commonly associated with a morbidity rate of up to 4%, but it is also associated with end-organ damage in the form of hypertensive emergency. As such, it is of utmost importance to diagnose the underlying cause and concomitantly treat hypertensive crisis. Although there is a paucity of information on the prevalence and incidence of hypertensive crisis, the potential dangers to neurologic, visual, cardiac, and renal structures are well-known. By understanding and further building upon the current knowledge on the etiology, diagnosis, and management of hypertensive crisis, in addition to utilizing novel technological breakthroughs, clinicians worldwide may further refine diagnosis, and management of hypertensive crisis in the pediatric population.

## Author Contributions

RR, ZM, AS, RC, SM, SS, GK, and DK contributed to the conception and design and wrote sections of the manuscript. All authors contributed to manuscript revision and read and approved the final manuscript.

## Conflict of Interest

The authors declare that the research was conducted in the absence of any commercial or financial relationships that could be construed as a potential conflict of interest.
